# Regional variation in caesarean deliveries in Germany and its causes

**DOI:** 10.1186/1471-2393-13-99

**Published:** 2013-05-01

**Authors:** Rafael T Mikolajczyk, Niklas Schmedt, Jun Zhang, Christina Lindemann, Ingo Langner, Edeltraut Garbe

**Affiliations:** 1 Department of Clinical Epidemiology, Leibniz Institute for Prevention Research and Epidemiology-BIPS, Bremen, Germany; 2 Department of Epidemiology, Helmholtz Centre for Infection Research, Inhoffenstr. 7, Braunschweig, 38124, Germany; 3 Department of Infectious Diseases Epidemiology, Hannover Medical School, Hannover, Germany; 4 MOE-Shanghai Key Laboratory of Children’s Environmental Health, Xinhua Hospital, Shanghai Jiaotong University School of Medicine and School of Public Health, Shanghai, China; 5 Faculty of Public Health, HFH Hamburg Distance University of Applied Sciences, Hamburg, Germany

**Keywords:** Caesarean section, Regional differences, Absolute and relative indications, Time trends

## Abstract

**Background:**

Determinants of regional variation in caesarean sections can contribute explanations for the observed overall increasing trend of caesarean sections. We assessed which mechanism explains the higher rate of caesarean sections in the former West than East Germany: a more liberal use of caesarean sections in the case of relative indications or more common caesarean sections without indications.

**Methods:**

We used a health insurance database from all regions of Germany with approximately 14 million insured individuals (about 17% of the total population in Germany). We selected women who gave birth in the years 2004 to 2006 and identified indications for caesarean section on the basis of hospital diagnoses in 30 days around birth. We classified pregnancies into three groups: those with strong indications for caesarean section (based on classification of absolute indications recommended by the Unmet Obstetrics Need network), those with moderate indications (other indications increasing the probability of caesarean section) and those with no indications. We investigated the percentage of caesarean sections among all births, presence of strong or moderate indications in all pregnancies, the probability of caesarean sections in the presence of indications and the fraction of caesarean sections attributable to strong, moderate and no indications.

**Results:**

In total, 294,841 births from 2004–2006 were included in the analysis. In the former West Germany, 30% births occurred by caesarean section, while in the former East Germany the caesarean section rate was 22%. Proportions of pregnancies with strong and moderate indications for caesarean section were similar in both regions. For strong indications the probability of caesarean section was similar in East and West Germany, but the probability of caesarean section among women with moderate indications was substantially higher in the former West Germany. Caesarean sections were also more common among women with no indications in the former West (8%) than in the former East (4-5%). The higher probability of caesarean section in the case of strong or moderate indications in the former West than in the East explained 87% of the difference between section rates in these two regions, while caesarean sections without indications contributed to only 13% of the difference observed.

**Conclusions:**

The observed difference between caesarean section rates in the former East and West Germany was most likely due to different medical practice in handling relative indications.

## Background

Caesarean section rates are on the rise worldwide [1-4]. The causes of this increase remain often hidden and can differ across countries. In Germany, substantial differences in caesarean section rates between the former eastern and western parts have been observed [5]. Absolute indications for caesarean section, which are responsible only for a small share of caesarean sections in developed countries, are unlikely to explain the partly large differences. But it remains unclear whether the differences arise from different medical practice (in such case either relative indications can be diagnosed more frequently, or in case of relative indications there is a higher probability of caesarean section being performed) or from different preferences regarding caesarean sections without indications. If caesarean sections are performed more commonly in the presence of relative indications, the difference may be attributable to medical practice. In contrast, if the difference arises from caesarean sections without indications, the woman’s or physician’s preference is likely to play a major role. We studied the components of East-West differences with regard to caesarean section by assessing: a) the prevalence of indications for caesarean section in all births, b) the risk for caesarean section in the presence of indications, c) the difference in caesarean section rates attributable to caesarean sections with and without indications.

## Methods

### Sample

We analysed data from the German Pharmacoepidemiological Research Database (GePaRD). The database has been described elsewhere [6-10]. In brief, GePaRD consists of claims data from four German statutory health insurances with more than 14 million people (around 17% of the total population) across Germany. The database contains in- and outpatient diagnoses, diagnostic and therapeutic procedures, and outpatient drug prescriptions. At the time of this analysis, data for 2004–2006 were available for all four health insurances included in GePaRD. The utilisation of health insurance data for scientific research is regulated by the Code of Social Law in Germany (SGB X). This study was conducted with permission from the Federal Ministry of Health, which is the responsible authority. Informed consent was not required, since the study was based on routinely collected anonymised data.

### Ascertainment of diagnoses and procedures

All hospital births to women 12 to 54 years old between January 1^st^ 2004 and December 31^st^ 2006 were identified using coding of diagnoses according to the International Classification of Diseases, German Modification, 10^th^ version (ICD-10 GM) and coding of operations and procedures (OPS). Births were classified as caesarean sections based on ICD-10 GM and OPS codes. To assess potential indications, we screened all admission and discharge diagnoses from hospitalisations starting in the 30 days before birth. Additionally, we included hospitalisations after birth if they ended within 30 days after birth. For codes indicating duration of pregnancy, we restricted the interval to 7 days before and after the delivery date, since ICD-10 codes indicating the duration of pregnancy (O09.-) can be used throughout the pregnancy i.e. also for admissions for reasons other than delivery.

The selection of diagnoses which can be potentially associated with a higher risk of caesarean sections was based on a review of the literature and content knowledge (the corresponding ICD-10 codes are provided in Table 1). Since the data does not contain the actual reason for caesarean section but only reimbursement diagnoses recorded around birth, the clinical classification of absolute and relative indications can be only approximated. Therefore the diagnoses were grouped into strong indications (corresponding to the classification proposed by the Unmet Obstetric Need network [11], but including also breech delivery among anomalies of foetal presentation) and moderate indications (all other diagnoses thought to increase the probability of caesarean section).

**Table 1:** Diagnoses with an expected higher risk of caesarean section and the corresponding ICD-GM 10 codes

**Diagnoses**	**ICD-GM 10 codes**
Twins and higher order pregnancies	O30, Z37.2-3, Z37.5-6, Z38.3, Z38.6
Anomalies of the foetal presentation	O32, O64
Intrauterine growth restriction	O36.5
Post date pregnancy	O09.7, O48
Preterm delivery	O09.3-5, O60.1, O60.3
Maternal distress	O75.0, O75.2, O75.3
Obstructed labour	O66
Asphyxia	O68
Prolonged birth	O63
Macrosomia	O36.6, O66.2
Placenta praevia	O44
Abruptio placentae	O45
Disproportion	O33
Anomalies of maternal pelvis	O34, O65
Intrapartal bleeding (excluded placenta previa)	O67
Failed induction	O61
Preexisting hypertonic disorders	O10
Non-severe hypertonic disorders during pregnancy	O11-13
Preeclampsia	O14
Eclampsia	O15
Complications because of umbilical cord	O69
Diabetes mellitus	O24.0-3
Gestational diabetes mellitus	O24.4
Uterine rupture	O71.0-1
Premature rupture of foetal membranes (PROM)	O42, O75.5-6
Abnorm contractions	O62.0-2
Previous caesarean section	O34.2

### Statistical analysis

We calculated the percentage of caesarean sections among all hospital deliveries stratified by year and federal state. To obtain representative numbers for Germany and regions of former West and East Germany (including Berlin), the proportion of caesarean sections per federal state was weighted by the total number of births in the corresponding federal state. Furthermore, we ascertained the prevalence of indications for caesarean section among all births. We then estimated the risk of caesarean sections among those with a specific indication. We used logistic regression to test for time trends and regional differences (East vs. West Germany), simultaneously adjusting for both sources of variation. Finally, we calculated the percentage of caesarean sections resulting from strong indications, moderate indications and no indications by maternal age and region. We also calculated the attributable fraction of caesarean sections for strong, moderate or no indications in East and West Germany. All statistical analyses were conducted with SAS 9.2 (SAS Institute Inc., Cary, NC). We specified the significance level at p < 0.01.

## Results

### Variation in caesarean section rates across federal states

**Figure 1:**
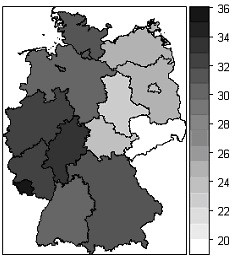
Variation in caesarean section rates across federal states in Germany (%).

In total, 294,841 births were included in the analysis (Table 2). Among them, 29% were caesarean sections. In 2004, the caesarean section rate in GePaRD was two percent points higher than in the hospital statistics for the whole of Germany, but the rates converged over time and in 2006 there was only one percent point difference. Across the federal states there were substantial regional differences, with caesarean section rates in the former West Germany at 30% and in the former East at 22%. In Berlin, the rate was close to those observed in the former East Germany despite the fact that territorially a larger part of Berlin belonged to the former West Germany. Within the former West and East Germany there was little variation across federal states (Figure 1).

**Table 2:** Percentage of caesarean sections among all hospital deliveries by year and federal states

	**GePaRD**			**Hospital statistics**		
	**2004**	**2005**	**2006**	**2004**	**2005**	**2006**
**All Births [n]**	100,160	99,195	95,486	682,767	664,597	652,642
Schleswig-Holstein	29.0	28.2	30.4	24.9	27.2	30.3
Hamburg	30.6	30.6	30.9	26.9	30.1	27.6
Lower Saxony	29.6	30.0	30.2	27.0	28.1	28.7
Bremen	27.8	26.4	26.3	28.8	26.1	29.5
North-Rhine Westphalia	30.6	31.4	32.1	27.9	28.6	29.6
Hesse	31.0	31.8	32.7	29.8	30.9	31.5
Rhineland-Palatinate	31.9	31.5	32.1	30.8	29.1	30.4
Baden-Württemberg	29.6	30.1	30.2	28.1	28.9	29.3
Bavaria	28.8	29.2	30.6	27.6	28.5	30.4
Saarland	33.2	31.4	34.4	33.6	32.1	33.2
Berlin	23.0	22.4	23.8	20.7	21.9	24.1
Brandenburg	22.4	23.3	25.0	20.6	21.3	23.6
Mecklenburg-Western Pomerania	25.6	26.4	24.8	23.0	23.4	24.7
Saxony	21.8	20.1	19.8	19.9	21.0	22.2
Saxony-Anhalt	23.0	22.7	23.1	20.6	22.2	22.5
Thuringia	22.7	21.0	23.7	24.7	23.2	23.9
Former West Germany*	30.0	30.4	31.2	28.1	28.8	29.8
Former East Germany*^,+^	22.8	22.2	22.9	21.2	21.9	23.3
Total*	28.7	28.9	29.6	26.8	27.6	28.6

* Weighted estimate using crude birth rates per federal state.

^+^ including Berlin.

**Figure 2:**
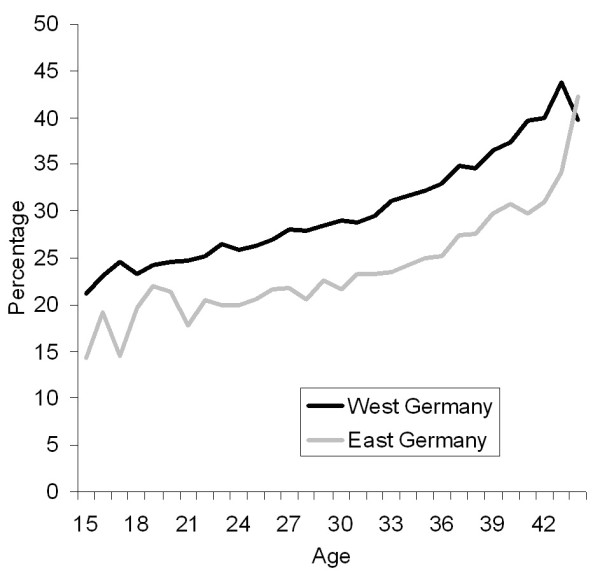
Percentage of deliveries by caesarean section by maternal age * and region (East and West Germany). * For graphical presentation age range was restricted to 15–44 years.

There was an almost linear increase in caesarean section rates by maternal age, with a doubling of rates from 20% to 40% between the ages of 15 and 44 in the former West and from 15% to 30% in the former East (Figure 2).

### Prevalence of indications for caesarean section in all births

Over the three years, there were no major changes in the prevalence of diagnoses providing indications for caesarean section, with the exception of the diagnosis of asphyxia which increased from 21 to 24% of all births in former East Germany (Table 3). However, because of several minor changes, the fraction of women with at least one indication for caesarean section increased by 2–3 percent points in both regions and correspondingly, the fraction of pregnancies without any indications decreased. For strong indications, there was no change over the study period. There were some differences between both regions, but most of them were less than one percent point. The overall percentages of women with at least one strong, at least one moderate and no indications for caesarean section were very similar in both parts.

**Table 3:** Prevalence of complications of pregnancy and labour with a higher risk for caesarean section by region and year (all hospital births in the GePaRD) (% with a given diagnosis)

	**Former East Germany**			**Former West Germany**			**By year**	**By region**
	**2004**^**a**^	**2005**	**2006**^**f**^	**2004**^**a**^	**2005**	**2006**^**f**^		
**Diagnoses**^**b**^	**%**	**%**	**%**	**%**	**%**	**%**	**p-value**^**e**^	**p-value**^**e**^
Twins and higher order pregnancies	1.86	1.64	1.64	2.02	1.73	1.92	0.0506	0.0101
Anomalies of the foetal presentation	9.42	9.81	9.57	9.71	9.85	10.12	0.0100	0.0497
Intrauterine growth restriction	3.33	3.49	3.88	3.39	3.69	4.19	<.0001	0.0376
Post date pregnancy	15.85	16.64	16.66	13.62	15.10	14.73	<.0001	<.0001
Preterm delivery	7.58	7.31	7.38	7.69	7.59	7.36	0.0155	0.3190
Maternal distress	0.55	0.50	0.71	0.47	0.51	0.41	0.5211	0.0005
Obstructed labour	1.15	1.01	1.02	1.39	1.61	1.45	0.6837	<.0001
Asphyxia	20.92	22.56	23.63	19.30	20.59	20.88	<.0001	<.0001
Prolonged birth	8.07	7.77	7.48	9.62	10.08	10.19	0.0087	<.0001
Macrosomia	1.57	1.69	1.79	1.33	1.38	2.17	<.0001	0.2974
Placenta praevia	0.36	0.41	0.49	0.52	0.51	0.51	0.5895	0.0086
Abruptio placentae	0.50	0.56	0.48	0.64	0.54	0.58	0.1963	0.0582
Disproportion	2.29	2.15	2.18	3.57	3.30	3.40	0.0506	<.0001
Anomalies of maternal pelvis	10.25	9.62	9.73	13.03	13.69	14.24	<.0001	<.0001
Intrapartal bleeding (excluding placenta previa)	0.34	0.30	0.46	0.39	0.58	0.56	<.0001	<.0001
Failed induction	1.00	0.94	0.98	0.95	1.19	1.31	<.0001	0.0006
Preexisting hypertonic disorders	0.39	0.44	0.44	0.31	0.33	0.34	0.2226	0.0013
Non-severe hypertonic disorders during pregnancy	3.76	3.83	3.79	3.06	3.34	3.39	0.0013	<.0001
Preeclampsia	3.21	3.32	3.19	3.04	2.99	2.89	0.1234	0.0015
Eclampsia	0.17	0.08	0.13	0.15	0.13	0.13	0.1504	0.5808
Complications because of umbilical cord	10.25	11.00	11.40	9.29	9.95	10.15	<.0001	<.0001
Diabetes mellitus	0.42	0.39	0.33	0.39	0.36	0.40	0.8311	0.9695
Gestational diabetes mellitus	1.97	2.28	2.45	3.44	3.72	3.77	<.0001	<.0001
Uterine rupture	0.18	0.15	0.21	0.28	0.26	0.26	0.4876	0.0005
Premature rupture of foetal membranes (PROM)	19.51	20.18	20.40	19.33	20.24	20.50	<.0001	0.9789
Abnorm contractions	6.33	6.97	6.51	4.89	5.41	5.22	0.0037	<.0001
Previous caesarean section	5.14	4.94	4.77	7.36	7.88	8.36	<.0001	<.0001
At least one strong^c^ indication for caesarean section	12.35	12.68	12.43	14.12	13.91	14.32	0.2773	<.0001
At least one of the moderate^d^ indications for caesarean section	58.33	60.06	60.98	56.65	59.71	59.99	<.0001	<.0001
Patients with no indication of diagnoses which could justify caesarean section	29.31	27.26	26.59	29.23	26.39	25.69	<.0001	0.0041

^a^ Restricted to births from February till December.

^b^ The same patient could have several of the listed diagnoses.

^c^ As strong indications were classified: *Placenta praevia*, *Abruptio placentae*, Uterine rupture, anomalies of foetal presentation and disproportion [11].

^d^ All indications listed in the table and not classified as strong were classified as moderate.

^e^ p-values obtained from logistic regression simultaneously adjusting for time trends and regional differences.

^f^ Restricted to births from January till November.

### Risk of caesarean section in the presence of indications

We also analysed how often caesarean sections were performed when specific conditions existed (Table 4). As expected, there was a large variation in the probability of caesarean section across different conditions. Indications classified as strong were associated with a probability of caesarean section of 75% or more. Additionally, the fraction of caesarean sections was high (>50%) in the case of twins or higher order pregnancies, anomalies of foetal presentation, disproportion or anomalies of maternal pelvis, failed induction, eclampsia, and previous caesarean section. In contrast, for some of the moderate indications the risk of caesarean sections was below 30%. For patients with no indications, the probability of a caesarean section was 4-5% in the East and 8% in the West.

**Table 4:** Probability of caesarean section given selected pregnancy and intrapartum conditions

	**Former East Germany**			**Former West Germany**			**By year**	**By region**
	**2004**^**a**^	**2005**	**2006**^**f**^	**2004**^**a**^	**2005**	**2006**^**f**^		
**Diagnoses**^**b**^	**%**	**%**	**%**	**%**	**%**	**%**	**p-value**^**e**^	**p-value**^**e**^
Twins and higher order pregnancies	71.85	71.38	68.28	75.30	74.42	74.34	0.3512	0.0118
Anomalies of the fetal presentation	76.72	74.41	75.83	79.15	79.56	78.83	0.4934	<.0001
Intrauterine growth restriction	47.96	44.55	42.88	52.91	51.58	50.67	0.0259	<.0001
Post date pregnancy	18.58	17.88	19.96	24.73	24.96	25.80	0.0303	<.0001
Preterm delivery	50.49	46.55	46.63	54.25	54.34	55.44	0.7540	<.0001
Maternal distress	46.67	39.33	45.22	57.63	60.29	55.81	0.6702	<.0001
Obstructed labour	54.01	52.51	49.40	53.54	54.39	54.73	0.8577	0.3510
Asphyxia	31.08	29.07	29.75	36.92	36.54	37.24	0.9664	<.0001
Prolonged birth	44.84	45.66	50.08	48.84	49.63	50.60	0.0030	0.0009
Macrosomia	48.82	45.97	46.58	54.38	57.74	48.67	0.0014	0.0011
Placenta praevia	93.22	82.19	82.50	87.95	86.68	87.47	0.3814	0.4586
Abruptio placentae	87.65	85.86	82.28	84.28	86.68	86.85	0.5091	0.8071
Disproportion	80.59	79.42	80.00	83.23	82.10	81.98	0.2338	0.0476
Anomalies of maternal pelvis	64.46	67.41	69.80	77.69	78.74	78.79	0.0023	<.0001
Intrapartal bleeding (excluding placenta praevia)	52.73	64.15	50.67	51.55	50.11	57.18	0.1732	0.5729
Failed induction	63.80	70.30	71.07	82.11	86.05	86.88	0.0029	<.0001
Preexisting hypertonic disorders	49.21	41.56	49.30	63.25	52.06	54.40	0.0932	0.0115
Non-severe hypertonic disorders during pregnancy	38.85	36.15	36.57	48.93	47.59	48.01	0.3590	<.0001
Preeclampsia	57.39	50.85	57.12	61.49	63.40	65.15	0.0278	<.0001
Eclampsia	67.86	57.14	57.14	68.75	77.14	69.15	0.8089	0.1219
Complications because of umbilical cord	16.06	15.25	17.48	18.79	18.78	20.04	0.0254	<.0001
Diabetes mellitus	50.00	55.07	58.49	60.21	56.70	59.79	0.7618	0.2409
Gestational diabetes mellitus	32.60	33.83	34.09	41.21	40.99	42.09	0.4453	<.0001
Uterine rupture	93.10	88.89	94.12	89.10	88.73	91.94	0.3597	0.5007
Premature rupture of foetal membranes (PROM)	21.98	19.04	19.66	24.97	25.03	25.21	0.6226	<.0001
Abnorm contractions	18.19	13.91	18.27	19.00	17.37	17.10	0.0618	0.1223
Previous caesarean section	68.94	71.18	76.32	80.64	81.15	82.45	0.0003	<.0001
At least one strong^c^ indication for caesarean section	77.96	75.51	76.71	80.32	80.31	79.86	0.2480	<.0001
At least one of the moderate^d^ indications for caesarean section	20.78	19.62	20.77	28.40	28.63	29.28	0.0080	<.0001
Patients with no indication of diagnoses which could justify caesarean section	4.04	4.26	4.82	8.17	7.91	8.19	0.5343	<.0001

^a^ Restricted to data from February till December.

^b^ The same patient could have several of the listed diagnoses.

^c^ As strong indications were classified: Placenta praevia, Abruptio placaente, uterine rupture, anomalies of foetal presentation and disproportion [11].

^d^ All indications listed in the table and not classified as strong were classified as moderate.

^e^ p-values obtained from logistic regression mutually adjusting for time trends and regional differences.

^f^ Restricted to births from January till November.

Most of the changes over time in the risk of caesarean section given the presence of indications did not reach statistical significance (Table 4). The few exceptions were prolonged birth and anomalies of maternal pelvis, failed induction and previous caesarean section, for which the risk of caesarean section increased by seven percent points between 2004 and 2006 in former East Germany, and macrosomia for which the risk of caesarean section decreased in both East and West, but the change was more pronounced in the West.

In contrast to the limited changes over time, there were substantial regional differences in the risk of caesarean section in the presence of indications (Table 4). Overall, in the presence of strong indications, there was no regional difference with respect to the risk of caesarean section – in contrast, the risk of caesarean section was 20% for women with moderate indications in the former East and 28% in the former West.

Given the percentage of pregnancies with indications and the probability of caesarean section in the case of existing indications, most of the difference in the section rates between the regions of East and West Germany was attributable to caesarean sections with indications (83%), while caesarean sections in pregnancies without indications contributed less to the difference (13%).

### Caesarean sections attributable to strong, moderate and no indications

**Figure 3:**
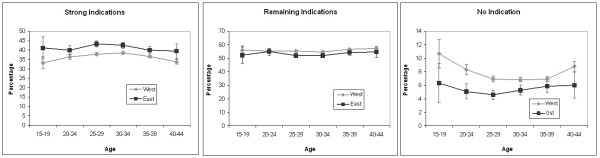
**Percentage of caesarean sections with i) at least one strong indication**^**a**^**, ii) with moderate indication(s), but no strong indications and iii) no indications by age and region. **^a^ As strong indications were classified: *Placenta praevia*, *Abruptio placaente*, Uterine rupture, anomalies of foetal presentation and disproportion [11].

The percentage of caesarean sections resulting from strong indications was slightly higher in the East than in the West (Figure 3). The percentages of of caesarean sections attributable to relative indications in the West and in the East were similar, while the percentage of caesarean sections in the absence of indications was higher in the West. There were more caesarean sections without identifiable indications among young women.

## Discussion

We found that the substantial difference in caesarean section rates between the former East and West Germany, 15 years after the reunification of Germany had two components. For a long list of conditions (moderate indications), the probability of caesarean section was considerably higher in the former West than in the East. The prevalence of these conditions did not substantially differ between the two former parts of Germany, which excludes the over-coding of potential justifications for caesarean sections as a potential explanation of the difference. Additionally, the probability of a caesarean section for women without any of the studied conditions was substantially higher in the West, but most of the difference in caesarean section rates between both parts was explained by a higher probability of caesarean sections in case of indications in the western part of Germany.

Extreme variations in caesarean section rates exist across countries worldwide, but in this study we examined the regional variation within one country. Substantial differences in caesarean rates between regions were also observed in other countries [12,13]. Our findings agree with the analyses of time trends in caesarean sections by demonstrating that the differences cannot be explained by clinical risk factors or maternal characteristics [14,15]. The interesting point about Germany is that while some variation within both former parts was also observed, the major difference was between them, which most likely means, that there are different traditions originating from the historical division of Germany.

There was nearly no difference in the prevalence of indications for caesarean section between the East and West. With most of the difference attributable to the higher probability of caesarean sections in the presence of indications, the different medical practice provides a likely explanation for the observed phenomenon, consistent with other studies [12,13]. The coexistence of caesarean section rates varying by 50% across different regions is astonishing, given that both East and West Germany have the same language, media, and social system. The training of medical doctors is standardised across Germany and there is an exchange among staff between regions. Still, in some way, different traditions persist in both former parts. Additionally, there is some contribution of caesarean sections without identified indications to the East-West difference. This fact is likely to reflect different preferences of the women, but also possibly more liberal opinions regarding caesarean sections without indications held by the doctors in the West.

Given the high probability of caesarean section conducted among women with a previous caesarean section (>70% in the East and >80% in West Germany), primary caesarean sections determine repeat caesarean sections in subsequent pregnancies. This mechanism is of concern as a potential cause of further increase in caesarean section rates [15]. Unfortunately, health insurance data in Germany does not contain information on parity. Given the lower fertility in the Eastern part in the 1990s [5], the fraction of primiparous women was likely higher in the East than in the West in the studied period, which should increase caesarean section rates in the East part i.e. the difference in parity-specific caesarean section rate would in reality be even larger. Although the average age at delivery is also lower in the East part [5], this variable does not explain the regional difference as can be seen in Figures 2 and 3 which are stratified by age.

Our findings on the subdivision of caesarean sections in those with strong, moderate and no indications underline once more the fundamental paradox of the optimal section rate. Only 35-40% of caesarean sections were conducted due to strong indications and for a majority of sections the indications were non-absolute. The non-absolute indications provide room for change. By linking different section rates with pregnancy outcomes it should be possible to address the question of the optimal section rate in further research.

### Strengths and limitations

The strength of the analysis is the use of the large database. While this database might not be representative of the entire population in all regions of Germany, it is likely to include the same segment of the population with respect to social status across different regions, making direct regional comparisons possible. A further strength is that we were able to include not solely diagnoses from the hospital stay during which birth occurred, but also diagnoses from hospitalisations around delivery time. This should minimize the effects of incomplete coding of diagnoses during the hospital stay ending with birth.

This study also has several important limitations. The database consists of claims data i.e. diagnoses were recorded for the purpose of reimbursement rather than for complete clinical or epidemiological assessment. Nevertheless, several diagnoses included in this analysis were medically unequivocal and for those a correct coding should be expected. German health insurance data does not contain information on body mass index which did not allow the inclusion of this risk factor in the analysis. We also did not establish which caesarean sections were conducted without the onset of labour (planned caesarean section) because a preliminary analysis demonstrated that the necessary information was only available in a small fraction of the cases. Furthermore, we did not assess absolute and relative indications for caesarean section as proposed by the guidelines, but only studied the presence of conditions which might increase the probability of caesarean sections and the occurrence of caesarean sections in women with a record of these conditions. Particularly, we were not able to separate breech delivery from other anomalies of fetal presentation, which resulted in classification of breech presentation as strong indication for caesarean section. Also other codes provided some room for misclassification. Formally assessing absolute and relative indications and causes of caesarean section did not appear possible without a standardisation of indications and without further information from medical records. A further limitation is that we only included indications related to obstetric outcomes and did not consider for example psychiatric codes. Failure to account for these cases might have increased the fraction of caesarean sections without indications, but this effect should be minor.

## Conclusions

To conclude, the substantial difference in caesarean section rates between both former parts of Germany, fifteen years after reunification, was most likely due to different medical practice in handling of pregnancies with relative indications for caesarean sections. These differences possibly originate from the past and persist despite a standardised training of the medical staff across Germany.

## Competing interests

RTM received research funding from Sanofi Pasteur and Bayer Pharma. NS, CL and IL participated in projects funded by Bayer Pharma. JZ has no conflict of interests. EG is running a department that occasionally performs studies for pharmaceutical industries. The companies include Mundipharma, Bayer Pharma, Stada, SanofiAventis, SanofiPasteur, Novartis, Celgene and GSK. EG has been consultant to Bayer Pharma, Nycomed, Teva and Novartis in the past.

## Authors’ contributions

RTM designed the research question and drafted the manuscript. NS conducted the analysis. NS and CL contributed sections of the manuscript. IL conceptualised parts of the analysis. JZ, IL and EG provided comments on the manuscript which substantially improved its content. All authors read and approved the final version.

## Pre-publication history

The pre-publication history for this paper can be accessed here:

http://www.biomedcentral.com/1471-2393/13/99/prepub
